# COVID-19 response in South African communities: Screening, testing, tracing and movement modelling

**DOI:** 10.7196/SAMJ.2022.v112i5b.16072

**Published:** 2022-05

**Authors:** M Modisenyane, L Madikezela, S Mngemane, O P Ramadan, M Matlala, K McCarthy, N Govender, T Nemungadi, S P Silal

**Affiliations:** 1 South African National Department of Health, Pretoria, South Africa; 2 Clinton Health Access Initiative, Pretoria, South Africa; 3 National Institute for Communicable Diseases, a Division of the National Health Laboratory Service, Sandringham, Johannesburg, South Africa; 4 World Health Organization, Nairobi, Kenya; 5 Modelling and Simulation Hub, Africa (MASHA), Department of Statistical Sciences, University of Cape Town, South Africa; 6 Nuffield Department of Medicine, Centre for Global Health and Tropical Medicine, Oxford University, UK

## Abstract

In South Africa (SA), the first case of COVID-19 was reported on 5 March 2020 from a traveller who had returned from Italy. Increases in COVID-19 cases and deaths necessitated the design and implementation of community screening, testing, and tracing as a control strategy. The SA government’s plans to implement community-based screening, testing, contact tracing and movement modelling during the early phases of the COVID-19 pandemic presented both opportunities and challenges. In this article, we present our experiences, opportunities and lessons for community-based COVID-19 response, anchoring these efforts in the primary healthcare system.

COVID-19 was first identified in December 2019 in Wuhan, China, and spread globally within weeks, resulting in an ongoing pandemic.^[1,2]^ In South Africa (SA), the first case of COVID-19 was reported on 5 March 2020 from a traveller who had returned from Italy.^[3]^ Within a space of 18 days, 402 cases were detected among people with no travel history, and clusters of cases were reported, followed rapidly by community transmission. The SA government swiftly implemented various control, prevention, containment, and mitigation measures to reduce transmission by introducing non-pharmaceutical interventions, including frequent hand washing, maintaining physical distance, use of masks, sanitising of hands using alcohol-based solutions, and quarantine.^[3,4]^ These strategies were implemented with the aim of reducing the rate of transmission of the virus, especially if implemented effectively at an early stage of the outbreak. Despite the early implementation of a range of interventions, the increase in COVID-19 cases and deaths in SA by 30 April 2020 suggested a need to review the design and implementation of community screening, testing, tracing, and movement modelling as a control strategy.

## Methods

This article represents a review of available literature and the real-time experiences, lessons learned, perspectives and expertise of the members of various teams, including the national epidemiological modelling, surveillance and response, community screening and testing (CST) and contact tracing workstream teams of the National Incidence Management Team. The objective is to highlight areas where community-based case finding, contact tracing and movement modelling could be utilised not only to provide a platform to ensure access to essential and routine healthcare services but also as a critical foundation for direct surveillance, response, and disease management and control of outbreaks.

### Community screening and testing

Aligned with the World Health Organization (WHO) principles of outbreak response, on 30 March 2020, the President announced a National State of Disaster, in terms of Section 27(5)(c) of the Disaster Management Act, 2002 (Act 57 of 2002), and included a series of risk-adjusted strategies and restrictions.

CST is one of the active-case-finding strategies adopted to facilitate early identification of probable COVID-19 cases, to support early referral for testing, to identify individuals who are sick and facilitate referral to health facilities, and to provide health education, prevention, and awareness on COVID-19 to promote behaviour change. When COVID-19 cases began to spread in various communities, the National Department of Health (NDoH) decided to mobilise community healthcare workers (CHWs), who were already working in communities providing health services, to conduct active COVID-19 case finding. CHWs and their supervisors, who are enrolled nurses known as outreach team leaders (OTLs), were redeployed in areas with confirmed COVID-19 cases to conduct community screening and specimen collection. More than 28 000 CHWs conducted home visits to undertake active house-to-house active-case finding.

CHWs were trained to use a standardised questionnaire to determine possible symptoms, exposure and risk, while OTLs collected specimens from all individuals with suspected COVID-19 infection. In areas where OTLs or professional nurses were not available, all individuals with suspected COVID-19 infections were referred to health facilities or mobile testing stations. The most dominant approach to CST was door-to-door screening, where every person in the household was screened using a mobile-phone application, and data regarding each household was uploaded to a central database.

**Fig. 1:**
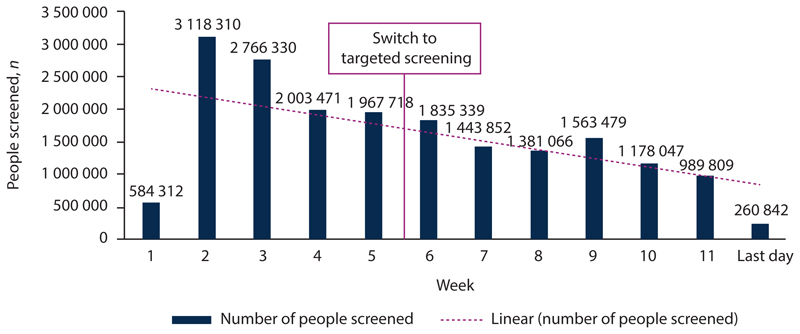
Cumulative community screening by week.

**Fig. 2:**
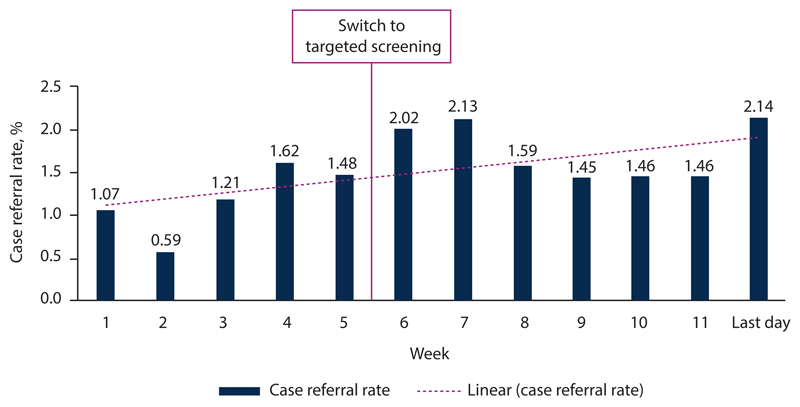
Community screening case finding by week.

SA used a combination of mass screening, targeted testing, and lockdown to control the early stages of the coronavirus outbreak that threatened to overwhelm the country. Mass screening of community members in ‘hotspot’ areas commenced on 4 April 2020 and by 24 May 2020 11 114 600 screenings had been conducted. With constrained testing challenges, as well as a lack of test reagent and viral extraction kits, SA changed its mass testing approach in July 2020 to focus on targeted screening and testing. A targeted testing strategy was implemented, which allowed provincial teams to: (*i*) identify which broad phase of the epidemic that area was currently experiencing; (*ii*) determine the general level of technical response activities such as surveillance, screening, laboratory testing, contact tracing, communications, etc.; and (*iii*) divert appropriate resources to the district or subdistrict level commensurate with the required level of technical response activities. The revised strategy included identifying hotspot areas for targeted interventions based on COVID-19 burden, screening the population in identified hotspot areas for COVID-19, and testing those individuals in outbreak and hotspot areas who screened positive as per the person under investigation (PUI) criteria, prioritising vulnerable groups. The aim was to align community testing with the National Health Laboratory Service (NHLS) testing capacity and to reduce test result turnaround times. At the time of introducing the targeted screening and testing strategy, the NHLS daily testing capacity, combined with the private sector laboratories, was between 10 000 and 15 000 tests per day. As at 4 February 2021, 1 497 075 NHLS tests were conducted through referral from CST with a 19.25% positivity rate, not that far from the national positivity rate of 24.56%, suggesting that community-level screening and referral for testing had a sufficiently high yield to justify its implementation as part of the control strategy. Figs 1 and 2 show the impact of implementation of the targeted testing strategy on the CST programme (decrease in number of people screened v. increase in case-finding rate).

Several challenges were encountered during the implementation of community screening and testing that hindered the effectiveness of the programme. These included:

- Fake news that added to anxiety and community hesitancy to allow CHWs into their homes.
- CST activities were largely documented and reported manually, resulting in delayed and interrupted reporting.
- Data collection was paper based, with inherent limitations that affected data quality, including multiple reporting lines.
- Multiple recommended mobile applications for screening and data reporting on CST activities led to poor uptake of the sanctioned NDoH mobile applications, leading to manual data collection and reporting.
- Human resource capacity challenges: the limited number of CHWs, OTLs and nurses deployed to cover all identified COVID-19 hotspot areas.

### Community contact tracing

SA used a decentralised contact tracing approach at the provincial, district and facility level. Dedicated contact tracer teams in the 52 districts were responsible for calling all the contacts of those diagnosed with COVID-19. They asked specific questions (e.g. date of the most recent contact, duration of their interaction, etc.) to investigate which ones were close contacts, to establish isolation measures or offer testing, if they had symptoms. While the decentralised approach facilitated outbreak proximity response to the population, it resulted in uneven contact tracing across and within provinces, given the existing geographical disparity in public health capacity and service provision. SA’s contact tracing approach combined manual contact tracing (using mobile phone calls, bulk short-messaging-system (SMS) texts, and home visits, where necessary), with technological solutions such as COVIDConnect self-services including an anonymised and decentralised contact tracing mobile application. However, privacy concerns led to a delay in the implementation of digital solutions. The COVIDConnect mobile application is used to self-administer a symptom checklist, and data for each household are uploaded to a central database to map screening coverage. People with COVID-19 symptoms are referred to mobile testing stations or nearby health facilities. As a result of privacy concerns, the uptake and scalability of the COVIDConnect self-surveillance have been very low.

**Fig. 3:**
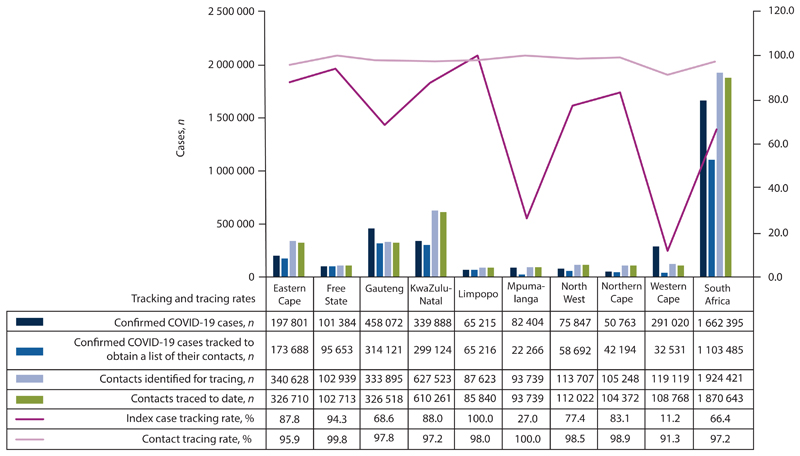
Case tracking and contacts tracing by province as at 31 May 2021. (Source: Provincial data as reported).

As at 31 May 2021, 5 of the 9 provinces in SA (Northern Cape, Limpopo, KwaZulu-Natal, Free State and Eastern Cape) performed above the national target rate of 80% for the index case tracing – which is a measure of coverage and timeliness of contact tracing (Fig. 3). Furthermore, Fig. 3 illustrates contacts traced/reached by tracer teams. This indicator measures the coverage of contact tracing by looking at the proportion of contacts reached and how quickly they are reached and quarantined. As seen in Fig. 3, all provinces performed above the national target of 90% for contacts traced. However, this performance is based on a low base, as only a small proportion of cases are diagnosed, and a small number of contacts are identified.

The challenges observed during the implementation of contact tracing included:

- Workforce challenges to manage the volume of contacts to be traced during the surge in cases.
- A low uptake of automated or digital contact tracing mobile application tools such as COVIDConnect self-service and COVID Alert mobile application.
- Only a small proportion of total cases and close contacts were being reached by contact tracers in time to prevent onwards transmission.
- Non-adherence to isolation and quarantine guidelines by index cases and close contacts.
- Testing skewed towards symptomatic cases, which are the minority.
- Not all symptomatic people test, and not all negative tests are true negatives.
- Insufficient administrative, material, and other logistics support, such as transportation, mobile phones, mobile phone airtime and data credit and laptops, to mount an effective contact tracing system.

### Modelling of community movement

The South African COVID-19 Modelling Consortium (SACMC) was established in March 2020 to project the spread of the disease to support policy and health system planning in SA. Several sets of projections and guidance documents were provided over the two waves of the epidemic. During the first wave, the National COVID-19 Epi Model (NCEM),^[5]^ a stochastic compartmental transmission model, was developed to estimate the total and reported incidence of COVID-19 in SA. The model followed a generalised Susceptible-Exposed-Infectious-Recovered (SEIR) structure accounting for disease severity (asymptomatic, mild, severe and critical cases) and the treatment pathway (outpatients, non-intensive care unit (ICU) and ICU beds). Several iterations of the model were produced as data became available. At the beginning of July 2020, the spatial scale of the model was extended from the 9 provinces to include the 52 districts in SA, reflecting the population size and connectivity between each district. Model calibration to hospital admissions and deaths were still computed at the provincial level, because of limited district-level hospital data at the time.

Movement and connectivity between districts were estimated based on aggregated mobile event data provided by Vodacom. District-to-district connectivity matrices were constructed based on the proportion of mobile phone pings that occurred in each district outside the home district. The home district was defined as the location where a mobile device is normally located between 22h00 and 04h00.

**Fig. 4:**
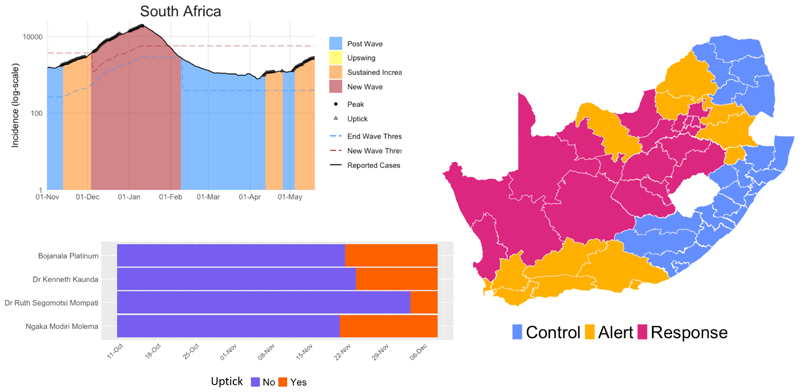
SACMC Epidemic Explorer,^[6]^ 21 May 2021. (SACMC = South African COVID-19 Modelling Consortium).

Summary connectivity matrices were constructed for each phase of lockdown (pre-lockdown, COVID-19 risk-adjusted strategy for level 5, level 4, level 3 (disaggregated) and level 2) to reflect the average movement between the 52 districts within each restriction period. The spread of SARS-CoV-2 between the districts and the trajectory of cases, admissions and deaths were made available to planners at the national, provincial and district level through an interactive dashboard. The outputs of the model were used to inform resource requirements and predict where gaps could arise based on the available resources within the SA health system. At the start of the second wave, there was much uncertainty surrounding the drivers of rapid increases in cases. To aid the detection of a resurgence at a rapid pace, the SACMC developed the SACMC Epidemic Explorer;^[6]^ a dashboard to explore the COVID-19 epidemic in SA, analysing resurgence risk, resurgence thresholds, presenting metrics to prepare for future outbreaks, and monitoring COVID-19 hospital admissions. This dashboard (both the secure and open-access versions) presents a subset of the metrics used to support the planning efforts of the SA government. Metrics on cases per population, growth in cases and sustained growth are presented at the district and subdistrict level. Through visual displays such as heatmaps, line plots and maps, with downloadable reports, the SACMC Epidemic Explorer rapidly became an invaluable resource tool for monitoring the trajectory of the epidemic throughout the country (Fig. 4).

The key challenge with modelling community transmission related to the spatial granularity of data. Provincial, district and subdistrict case allocations were mapped primarily according to a residential address, or the facility where the sample was collected. Missing data and procedures for extracting and geocoding address data could result in allocation errors, particularly at more granular spatial resolutions (i.e. subprovince level). While provincial allocations of cases were complete, district, subdistrict and ward data were available for only ~86%, 73% and 45% of cases, respectively.

## Discussion and recommendations

### Surveillance, data management and information systems

With ongoing waves of COVID-19, there is, therefore, a need to leverage existing primary healthcare (PHC) disease surveillance systems and databases to allow for a rapid and cost-effective transition towards COVID-19 surveillance.^[7]^ SA should quickly move away from manual case investigation and contact tracing systems and consider utilising digital systems such as the Global Influenza Surveillance and Response System, the new DHIS2 package for COVID-19 and Go.Data to identify populations for active-case finding and contact tracing, analyse the distribution of identified cases, and facilitate the referral of patients to linked facilities. While screening, testing, case investigation and contact tracing during the initial phase of the pandemic were justified to contain the pandemic, there is a need to refocus SA’s public efforts and resources, with an emphasis on targeting in higher-risk congregated settings.^[8]^ Some of the key public health strategies may include: strong messaging about mask wearing; conducting outbreak investigations and targeted case investigations; targeting prevention strategies to the most vulnerable persons, populations and settings; instituting and improving environmental health measures, etc. The development of the SACMC Epidemic Explorer, a dashboard to explore the COVID-19 epidemic in SA, has been instrumental in supporting decision-making, to determine how and when the epidemic was spreading spatially within the country. It will be important to sustain these dashboards in the wake of future waves.

### Leadership and governance

There is a need to establish an integrated national surveillance system that is adapted and strengthened at different levels of the health system to facilitate effective community screening, testing, case investigation and contact tracing efforts for COVID-19 in SA. There is therefore a need to establish a productive relationship between all levels of government, both decision makers and implementers, especially during a time of emergency central powers.^[9]^ There is a need to align surveillance strategies of community-based PHC, including ensuring that service delivery approaches consider population health needs and PHC system capacities linked to the implementation of COVID-19 efforts. By ensuring the involvement of communities, civil society, and the private sector, efforts to monitor and slow down the spread of COVID-19 could be multiplied.

### Workforce

Ramping up screening, testing and contact tracing in SA has led to the activation of well-co-ordinated and effectively led national rapid-response teams, including changing the roles of the existing workforce through task-shifting, re-assignment, changes to workforce rostering and recruiting of additional personnel. Although routine health services have been disrupted during the COVID-19 pandemic,^[10]^ COVID-19 has provided an opportunity for positive, long-term, systematic change to transform the current policies and practices.^[11]^ It is important that SA effectively integrates its strong cadre of CHWs into its health system and builds on their local knowledge. Efforts should be made to ensure that any new workforce hired to support COVID-19 activities are integrated into care teams to maximise co-ordination and continuity of care. SA’s mass screening, and later targeted screening and testing programme, during the early phases of the pandemic, was a helpful epidemiological surveillance tool, which was based on accumulative experiences from the existing Ward-Based Outreach Team (WBOT). Furthermore, by combining multiple mobility/movement data sources in an analysis, SA has been able to achieve an understanding of transmission patterns within its borders.

## Conclusion

SA has been able to develop and deploy a community-based screening, testing and contact tracing system to mitigate the spread of the virus during initial phases of the pandemic. However, with an increase in the proportion of people with some immunity from infection and/or vaccination, new knowledge about the epidemiology of the virus and the emergence of the more infectious Omicron variant, SA should adapt its response from control to mitigation strategies. Movement modelling was used to develop tailored risk communication campaigns to identify hotspots to educate the population about possible symptoms, testing requirements and the risk of COVID-19 transmission. The implementation of mitigation strategies should be focused on the most severe outcomes of COVID-19: hospitalisation and deaths. While it may not be optimal to investigate and monitor all individual COVID-19 cases and close contacts, case investigation and contact tracing still remain necessary public health tools for interrupting ongoing transmission of COVID-19 in the most vulnerable populations, especially in certain higher-risk congregated settings.
